# Rheumatoid Arthritis: Biomarkers and the Latest Breakthroughs

**DOI:** 10.3390/ijms262110594

**Published:** 2025-10-30

**Authors:** Meilang Xue, Hui Wang, Frida Campos, Christopher J. Jackson, Lyn March

**Affiliations:** Sutton Arthritis Research Laboratory, The Australian Arthritis and Autoimmune Biobank Collaborative (A3BC), Sydney Musculoskeletal Health, Kolling Institute, Faculty of Medicine and Health, The University of Sydney at Royal North Shore Hospital, St Leonards, NSW 2065, Australia; hwan0780@uni.sydney.edu.au (H.W.); fac2134@columbia.edu (F.C.); chris.jackson@sydney.edu.au (C.J.J.); lyn.march@sydney.edu.au (L.M.)

**Keywords:** rheumatoid arthritis, biomarkers, inflammation, multi-omics, synovial tissue

## Abstract

Rheumatoid arthritis (RA) is a heterogeneous autoimmune disease characterized by variable clinical manifestations and a complex, often unpredictable disease trajectory, which hinders early diagnosis and personalized treatment. This review highlights recent breakthroughs in biomarker discovery, emphasizing the transformative impact of multi-omics technologies and deep profiling of the synovial microenvironment. Advances in genomics and transcriptomics have identified key genetic variants and expression signatures associated with disease susceptibility, progression, and therapeutic response. Complementary insights from proteomics and metabolomics have elucidated dynamic molecular patterns linked to inflammation and joint destruction. Concurrently, microbiome research has positioned gut microbiota as a compelling source of non-invasive biomarkers with both diagnostic and immunomodulatory relevance. The integration of these diverse data modalities through advanced bioinformatics platforms enables the construction of comprehensive biomarker panels, offering a multidimensional molecular portrait of RA. When coupled with synovial tissue profiling, these approaches facilitate the identification of spatially resolved biomarkers essential for localized disease assessment and precision therapeutics. These innovations are transforming RA care by enabling earlier detection, improved disease monitoring, and personalized treatment strategies that aim to optimize patient outcomes.

## 1. Introduction

Rheumatoid arthritis (RA) is an autoimmune disease characterized by persistent inflammation, ongoing joint damage, and various systemic manifestations. Worldwide, RA affects 0.5% to 1% of the population, with a noticeably higher prevalence in women [1]. Although the precise cause of RA remains only partly understood, current evidence suggests a multifactorial interplay of genetic predisposition, environmental factors, and hormonal influences in the onset and progression of the disease [2].

RA is broadly divided into seropositive and seronegative categories, based on the presence or absence of autoantibodies, primarily rheumatoid factor (RF) and anti-citrullinated protein antibodies (ACPA). These subtypes have different genetic backgrounds, clinical features, immunopathological profiles, radiographic progressions, and prognoses [3]. Seropositive RA, identified by detectable RF and/or ACPA, benefits from well-established diagnostic and prognostic biomarkers. Conversely, seronegative RA, which accounts for about 15% to 25% of cases, presents a significant diagnostic and treatment challenge due to the lack of these serological markers. 

RA remains incurable but is clinically manageable with conventional synthetic and biologic disease-modifying antirheumatic drugs (cs/bDMARDs). Despite therapeutic advances, up to 40% of patients exhibit suboptimal responses or develop pharmacologic resistance, leading to refractory RA [4]. Long-term DMARD use, especially methotrexate, is linked to significant adverse effects, including gastrointestinal, pulmonary, and hematologic toxicity [5].

Early therapeutic intervention, ideally within 3 to 6 months of symptom onset, has been shown to improve remission rates and long-term outcomes [6]. The preclinical phase of RA, characterized by silent autoimmunity, can precede overt symptoms by several years. Its heterogeneity and lack of predictive tools for early diagnosis and treatment selection hinder timely intervention, increasing the risk of irreversible joint damage. There is a critical need for robust biomarkers to enable early diagnosis and inform personalized DMARD selection, especially in refractory RA. This review explores both established and novel biomarkers, highlighting the role of advanced technologies in identifying potential biomarkers in precision medicine for managing RA.

## 2. Current Biomarkers in RA

A biomarker is a measurable biological characteristic that indicates a normal or abnormal process, condition, or disease. In RA, current biomarkers predominantly include protein-based, genetic, and epigenetic signatures, most of which are detected in peripheral blood. Furthermore, imaging biomarkers obtained from modalities like ultrasound and magnetic resonance imaging (MRI) provide crucial structural and functional insights. These biomarkers support diagnostic and prognostic assessments, enable prediction of disease course, and inform therapeutic response and drug toxicity monitoring [7]. Their integration into clinical practice has significantly improved diagnostic accuracy and facilitated more personalized, outcome-focused treatment approaches.

### 2.1. Protein Biomarkers

#### 2.1.1. Autoantibodies

Autoantibodies are crucial for the development and clinical management of RA and are important in its diagnosis and progression. RF and ACPA often appear before symptom onset and are associated with more severe disease, forming part of the ACR/EULAR 2010 diagnostic criteria [8]. Additional autoantibodies, including anti-carbamylated protein (anti-CarP) [9] and anti-peptidyl arginine deiminase 4 (anti-PAD4) [10], further improve diagnostic and prognostic accuracy. Table 1 outlines the key autoantibodies associated with RA, including their sensitivity, specificity, prevalence in other conditions, and clinical applications.

Emerging data indicate that multiplex autoantibody profiling improves the prediction of treatment response. Co-expression of RF and ACPA is associated with better outcomes to tumor necrosis factor inhibitors (TNFi), while anti-CarP and anti-PAD4 may indicate a poorer therapeutic response [11]. A broader autoantibody repertoire may indicate increased immune activation [12,13], potentially boosting responsiveness to anti-inflammatory treatments. 

#### 2.1.2. Inflammatory Biomarkers

Inflammatory biomarkers, or acute-phase reactants, constitute a critical component in the diagnostic stratification, longitudinal monitoring, and therapeutic evaluation of RA. Principal analytes include C-reactive protein (CRP) and erythrocyte sedimentation rate (ESR) [14], serum amyloid A (SAA) [15], matrix metalloproteinase (MMP)-3 [16], calprotectin (S100A8/S100A9) [17], and 14-3-3η protein [18]. Additionally, key pro-inflammatory cytokines, such as interleukin (IL)-6 and TNF-α, play pivotal roles in RA pathophysiology and are increasingly leveraged as both biomarkers and therapeutic targets. Table 2 delineates their respective diagnostic accuracy, specificity profiles, and clinical applicability. 

Despite their utility, these inflammatory biomarkers exhibit elevated levels across a spectrum of inflammatory and autoimmune disorders, thereby limiting their disease-specificity. Accordingly, their optimal use lies in integrative diagnostic frameworks that combine inflammatory biomarkers with autoantibody panels and clinical phenotyping.

### 2.2. Genetic Biomarkers

RA pathogenesis is largely driven by genetic predisposition, with heritability estimates approaching 60% [19]. The genetic contribution is particularly pronounced in seropositive RA [20], where strong associations have been established with the *HLA*-*DRB104* and *DRB110* alleles [21]. In contrast, *HLA* associations in seronegative RA are comparatively attenuated. However, haplotypes such as *HLA-B08/DRB103* [22], as well as non-HLA variants including single-nucleotide polymorphisms (SNPs) in *CLYBL* and *ANKRD55* [23,24] have emerged as potential contributors to disease susceptibility. Table 3 lists some key genetic markers in RA, including their sensitivity, specificity, presence in other conditions, and clinical applications.

Variants within the JAK/STAT signaling pathway have been implicated in seropositive RA [25]. Notably, therapeutic response to JAK inhibitors appears to be independent of autoantibody status [26,27], suggesting a broader applicability across RA subtypes. Additional loci with established roles in RA pathogenesis include *CTLA4*, *TRAF1/C5*, *PTPN22*, and *PADI2/4*, each exhibiting differential allele frequencies and functional relevance across serotypes and populations. 

The polygenic nature of RA, compounded by complex gene-environment interactions, such as tobacco exposure, gut microbiota dysbiosis, and epigenetic modifications, poses substantial challenges in pinpointing causal genetic variants. Individual genetic markers frequently exhibit limited diagnostic sensitivity and specificity, emphasizing the need for integrative approaches that account for the multifactorial nature of disease susceptibility.

### 2.3. Epigenetic Biomarkers 

Epigenetic regulation, including DNA methylation, post-translational histone modifications, and non-coding (nc)RNAs, serves as a critical interface between genetic predisposition and environmental exposures in the pathogenesis of RA. These mechanisms orchestrate immune cell differentiation, modulate pro-inflammatory signaling cascades, and influence synovial tissue remodeling, positioning them as promising avenues for biomarker discovery [28,29,30].

Aberrant methylation patterns have been identified in peripheral T and B lymphocytes as well as synovial fibroblasts, even in early-stage RA [28], and are predictive of differential responsiveness to DMARDs [29]. Circulating methylation levels of genes such as *CXCR5* and *HTR2A* correlate with disease activity and may serve as minimally invasive biomarkers [31,32]. Histone acetylation dysregulation within synovial fibroblasts promotes transcriptional upregulation of IL-6 and MMPs, exacerbating local inflammation and joint destruction [33,34,35]. Pharmacologic inhibition of histone deacetylases (HDACs) has demonstrated therapeutic potential in preclinical models, underscoring HDACs as viable drug targets [36]. Beyond acetylation, additional histone modifications, including phosphorylation, ubiquitylation, and sumoylation, contribute to the dynamic epigenetic landscape of RA [37].

Integrating epigenomic profiling into clinical workflows may enhance early diagnostic accuracy, facilitate molecular stratification, and support the development of personalized treatment regimens. As such, epigenetic mechanisms represent a frontier in the advancement of precision medicine for RA.

### 2.4. Imaging Biomarkers

Imaging biomarkers are essential tools in RA management. They provide objective measures that significantly enhance early diagnosis, prognosis, and monitoring of treatment response. These biomarkers offer insights that go beyond conventional clinical indices, such as the DAS28 and ESR/CRP [38,39]. The most sensitive modalities for visualizing active joint inflammation are Musculoskeletal Ultrasound (MSUS) and MRI [40]. 

MSUS is a high-resolution, accessible, and dynamic imaging technique that is vital for initial diagnosis, especially in cases of undifferentiated or seronegative arthritis [41]. It is particularly useful for identifying subclinical synovitis and bone erosions that are often overlooked during physical examinations and conventional X-rays [39]. MSUS provides clear images of soft tissues, cartilage, and bone, allowing for the effective identification of early RA signs, including synovial hypertrophy and bone erosions [41]. Its role in guiding clinical decision-making is vital, especially in evaluating synovial inflammation and identifying patients at increased risk of relapse [41]. Notably, studies have demonstrated that patients in clinical remission with residual synovitis visible on MSUS are significantly more likely to experience flare-ups if their medication is tapered [42].

MRI is considered the most sensitive technique for detecting early inflammatory and destructive changes in RA [42,43,44]. It provides superior visualization of both soft tissue and bone structures, making it a valuable tool for early diagnosis and monitoring disease progression [42,43,44]. Key MRI findings include synovitis, bone marrow edema (BME), and erosions [38]. The presence of BME on MRI is especially important, as it signifies a more aggressive disease course and an increased risk of joint damage progression [42]. This insight frequently encourages rheumatologists to initiate early and assertive Treat-to-Target strategies [42]. Moreover, in cases of suspected RA, MRI-detected BME can predict the future development of RA, and in established early RA, it can predict later structural damage progression [45].

Other modalities also have specialized utility despite the dominance of MRI and MSUS in clinical practice. Recent advancements in positron emission tomography/computed tomography (PET/CT) scans are emerging as useful tools for monitoring disease activity and treatment response, providing a functional assessment of inflammation [46]. CT scans offer superior detail of bone structures compared to MRI, making them especially effective for visualizing bony erosions [38]. Interestingly, ultra-low-dose CT has been investigated for its potential to detect synovitis and may identify more differential diagnoses than MRI in patients with suspected RA [47].

Incorporating advanced imaging techniques into clinical practice offers significant diagnostic and prognostic benefits. However, challenges such as high MRI costs, limited accessibility, and the necessity for standardized training have restricted their use in practice [48]. Overcoming these barriers is crucial for maximizing the potential of imaging biomarkers in the personalized management of RA.

### 2.5. Limitations of Classical Biomarkers

Conventional biomarkers in RA, such as RF and ACPA, often lack clinical utility due to limited specificity and sensitivity. These markers can be present in other autoimmune or inflammatory conditions and are absent in a significant subset of RA patients, particularly in early or seronegative cases. Moreover, they offer minimal insight into disease heterogeneity, failing to capture the dynamic and localized nature of joint pathology [49].

This lack of precision hampers accurate diagnosis, stratification, and prediction of treatment response. As a result, reliance on single biomarkers leads to oversimplified assessments that do not reflect the complex immunopathology of RA [50]. Overcoming these limitations requires a shift toward multi-dimensional biomarker frameworks that integrate molecular, cellular, and tissue-level data for a more robust and individualized approach to disease management.

## 3. Breakthroughs in the Discovery of RA Biomarkers

Advances in multi-omics technologies are accelerating biomarker discovery in RA by integrating genomics, transcriptomics, proteomics, epigenomics, metabolomics, and microbiomics data with clinical parameters and machine learning (ML). High-throughput platforms such as next-generation sequencing (NGS) and mass spectrometry enable comprehensive molecular profiling, supporting earlier diagnosis, refined prognostication, and personalized therapy [51].

Synovial tissue analysis complements systemic profiling by offering direct insight into local inflammatory mechanisms. Multi-omics interrogation of synovial biopsies has identified molecular subtypes, predicted therapeutic responses [52], and improved diagnostic clarity in undifferentiated arthritis [46]. Though not yet standard practice, synovial profiling holds promise for advancing precision medicine in RA [53,54,55].

### 3.1. Multi-Omics Approaches 

#### 3.1.1. Genomics 

Genomics research has substantially advanced our understanding of RA, identifying both *HLA* and non-*HLA loci* that influence disease susceptibility, progression, and serological heterogeneity [56]. High-throughput platforms, including genome-wide association studies (GWAS), whole-genome sequencing, and SNP arrays, have identified over 100 loci, notably within *HLA-DRB1* and genes regulating immunity, inflammation, and signal transduction. These discoveries have elucidated the genetic architecture of ACPA-positive RA and its divergence from seronegative forms [22].

Beyond the major histocompatibility complex, non-HLA genes such as *ATXN2L*, *MMP-14*, *GALNT12*, and *KCNN2* are implicated in joint destruction and comorbidities [57,58,59], with Mendelian randomization and transcriptomic profiling supporting their translational relevance. Notably, IL-17 signaling [60] and *angiopoietin-2* polymorphisms [61] have emerged as pivotal in RA pathogenesis.

Population-specific GWAS underscore the necessity of inclusive genomics; studies in Chinese cohorts revealed novel loci (e.g., *IL12RB2*, *CCR2*) [62], while multi-ancestry meta-analyses uncovered 124 markers, 34 previously unreported [63]. These findings highlight ethnic variability in genetic risk.

Genomic insights also inform RA subphenotyping. A large GWAS (>31,000 cases) identified JAK/STAT pathway genes as central to seropositive RA, though seronegative RA remains genetically undercharacterized [25]. Collectively, these advances are propelling precision medicine in RA, enabling earlier diagnosis, individualized treatment, and preventive strategies. 

#### 3.1.2. Epigenomics 

Epigenomics, the study of heritable, reversible modifications to the genome that do not alter DNA sequence, is increasingly recognized as a critical dimension in RA research. Key epigenetic mechanisms, including DNA methylation, histone modifications, and non-coding(nc)RNAs, offer promising avenues for biomarker discovery, patient stratification, and personalized therapeutic strategies [64,65,66].

DNA methylation profiles are particularly valuable due to their stability and accessibility in easily obtainable samples like peripheral blood mononuclear cells and whole blood [28]. This stability makes them robust indicators for disease stratification and prognosis. Research has shown that specific CpG methylation signatures in peripheral blood can differentiate responders from non-responders to TNFi therapy [67] and predict progression from undifferentiated arthritis to RA [68]. A seven differentially methylated positions signature has shown utility in forecasting Leflunomide response [69], while methylation changes in *TRIM15*, *SORC2,* and *STAT3* correlate with methotrexate efficacy [70].

To address the challenges posed by large-scale analysis, targeted studies focusing on the methylation status of specific individual genes have provided significant and actionable insights for RA. Notably, altered methylation patterns in the *Absent in melanoma 2* (*AIM2*) gene, an essential component of the inflammasome pathway, have been observed in RA patients compared to healthy controls [71]. This finding suggests a connection between epigenetic regulation and inflammation mediated by the *AIM2* gene. Additionally, hypomethylation of *Homeodomain Interacting Protein Kinase 3* (*HIPK3*) found in peripheral blood is associated with RA and shows a negative correlation with inflammatory markers, such as CRP [72,73]. This highlights the potential of *HIPK3* as a diagnostic biomarker. Overall, these results underscore the promise of DNA methylation markers as non-invasive tools for early diagnosis, prognosis, and therapeutic stratification in RA.

Histone modifications, such as acetylation, methylation, and citrullination, play a crucial role in modulating chromatin architecture and gene expression in RA [74]. Specifically, the enrichment of trimethylation on histone H3 at lysine 4 in synovial fibroblasts serves as a marker for active promoters associated with pro-inflammatory gene expression [75]. Additionally, ncRNAs, including micro (mi)RNAs and long nc (lnc)RNAs, are increasingly recognized for their role in fine-tuning immune signaling pathways and are emerging as valuable diagnostic and prognostic tools for RA.

Environmental exposures such as smoking and pollutants influence epigenetic regulation. Methylation at cg21325723 mediates the interaction between rs6933349 and smoking, modulating ACPA-positive RA risk [76]. Gut microbiota-derived short-chain fatty acids (SCFAs) also impact on the histone deacetylase activity, promoting Treg differentiation and immune tolerance [77,78]. Longitudinal epigenomic studies are mapping methylation trajectories in at-risk individuals, revealing early predictive loci and dynamic changes in immune-related pathways during treatment [66,79]. Early-life exposures, e.g., maternal diet, stress, and neonatal factors, may induce lasting epigenetic changes that elevate RA susceptibility [80].

Combining DNA methylation and histone data with ML models is being developed to predict drug responses [81]. The integration of epigenetic markers with conventional biomarkers, such as RF and ACPA, enhances diagnostic accuracy, particularly for seronegative RA [82]. These advances underscore the potential of epigenomic profiling to refine RA diagnosis, forecast treatment outcomes, and guide precision medicine approaches. 

#### 3.1.3. Transcriptomics

Transcriptomics, the comprehensive analysis of RNA transcripts, is a critical tool for biomarker discovery and mechanistic insight in RA. By capturing dynamic gene expression profiles, transcriptomic approaches facilitate disease stratification and support precision medicine. Advances in bulk RNA sequencing (RNA-Seq), single-cell (sc)RNA-Seq, and spatial transcriptomics have enabled high-resolution mapping of transcriptomic activity across distinct cell types and tissue microenvironments [29].

Transcriptomic biomarkers encompass both protein-coding genes and diverse classes of ncRNAs. Differential expression of genes such as *ADAMDEC1* distinguishes RA from osteoarthritis [83], while SAA4 correlates with disease activity [84]. Alternative splicing events, including those affecting *CD44* and *survivin genes*, generate isoforms linked to joint destruction and disease progression [85] offering novel diagnostic and therapeutic targets.

NcRNAs play a pivotal role in RA pathogenesis by modulating inflammatory and matrix-degrading pathways [86,87,88]. MiRNAs, such as miR-146a and miR-155, are consistently dysregulated in RA and correlate with disease activity [82]; miR-499 polymorphisms are associated with susceptibility [89]. lncRNAs, including HOTAIR and NEAT1, influence fibroblast-like synoviocytes and immune cell function [90], contributing to chronic inflammation [91]. Circular (circ)RNAs, such as circPTPN22 [92], act as miRNA sponges [93,94] and are emerging as promising biomarkers for diagnosis and patient stratification.

Transcriptomic profiling has also identified early-stage RA biomarkers, such as CXCL10, MMP-3, IL-6, TNF, and S100A8/S100A9, that support early diagnosis and disease monitoring. Moreover, blood-based transcriptomic signatures have demonstrated predictive value for therapeutic response to TNFi, enabling more informed treatment decisions [95].

Collectively, transcriptomics offers a powerful framework for elucidating RA pathogenesis, identifying clinically actionable biomarkers, and guiding individualized therapeutic strategies.

#### 3.1.4. Proteomics 

Proteomics provides a comprehensive analysis of protein composition, interactions, and post-translational modifications [96], offering critical insights into molecular and cellular mechanisms underlying various autoimmune diseases. Advanced technologies such as Liquid chromatography-tandem mass spectrometry (LC-MS/MS), matrix-assisted laser desorption/ionization mass spectrometry (MALDI-TOF MS), Two-dimensional gel electrophoresis, and isobaric tagging (e.g., iTRAQ) [97] enable high-throughput quantification and characterization of protein profiles. Mass spectrometry remains central to proteomic workflows and is often integrated with immunoassays like enzyme-linked immunosorbent assay and cytometric bead arrays for validation [98].

Proteomics has emerged as a powerful platform for identifying diagnostic and prognostic biomarkers in RA. Recent studies have demonstrated its utility in detecting early-stage RA before clinical diagnosis by profiling serum analytes such as collagen triple helix repeat containing 1 [99,100], pregnancy zone protein, vitamin D binding protein (VDBP), SAA [84], and cytokines. Sirtuin 1 [101] and SAA4 have also shown promise in distinguishing RA from healthy controls and other inflammatory conditions.

Mun et al. [84] employed LC-MS/MS to identify serum proteins (Angiotensinogen, SAA4, VDBP, Retinol-binding protein 4) that differentiate RA patients, including seronegative cases, from controls. Additionally, integrative approaches combining proteomics with ML and protein–protein interaction networks have revealed 24 candidate biomarkers [102] with potential clinical relevance.

Methodological innovations continue to expand proteomic capabilities. Optimization of the Sequential window acquisition of all theoretical mass spectra (SWATH-MS) workflow has improved data reproducibility and proteome coverage, enabling more detailed plasma profiling. Targeted proteomics of synovial fluid has further elucidated post-translational modifications in autoantigens, offering new avenues for biomarker development and disease activity monitoring.

#### 3.1.5. Metabolomics

Metabolomics, which involves the comprehensive profiling of small-molecule metabolites, provides a high-resolution overview of systemic physiology and is gaining prominence in RA research. Notably, metabolic signatures, especially those originating from the gut microbiome, are being recognized as significant contributors to the pathogenesis of RA [103]. This emerging understanding holds promise for applications in early diagnosis, disease monitoring, and therapeutic stratification [104]. Table 4 presents a summary of various metabolites, highlighting their diagnostic performance, specificity, and potential clinical applicability as biomarkers for RA.

Microbial metabolites have demonstrated strong associations with RA-related inflammatory pathways [103,105]. SCFAs, such as butyrate and propionate, produced via microbial fermentation of dietary fibers, exert anti-inflammatory effects. Their reduced levels in RA patients suggest a role in promoting systemic inflammation and disease progression. Indole-3-propionate (IPA), a tryptophan-derived microbial metabolite, has been inversely correlated with RA risk [106], indicating a protective immunomodulatory function.

Sphingolipids, including sphingomyelin species like SM 16:1, are integral to membrane structure and signaling. These lipids have been linked to reduced RA susceptibility [106]. Heptadecasphin-4-enine, another sphingolipid, has shown diagnostic potential [107], reinforcing the relevance of lipid metabolism in RA. Similarly, Glycine, an anti-inflammatory amino acid, has been negatively associated with RA incidence [108]. Conversely, elevated argininosuccinate, an intermediate in the urea cycle and nitric oxide synthesis, is associated with increased RA risk [106], likely due to its pro-inflammatory properties.

Although current findings are largely correlational, they provide mechanistic insights into metabolic dysregulation in RA and highlight novel therapeutic targets. Strategies such as modulating microbial-derived metabolites or supplementing protective compounds may offer new avenues for intervention. 

#### 3.1.6. Microbiomics 

The human microbiota is a diverse ecosystem of microorganisms, including bacteria, archaea, fungi, and viruses, that plays a critical role in immune regulation, metabolism, and host homeostasis. Recent studies increasingly suggest that gut dysbiosis, which refers to an imbalance in these microbial communities, may be a key factor in the development of RA [109]. This disruption of microbial equilibrium often occurs before the appearance of clinical symptoms and can cause inflammation in the intestines, thereby impairing the integrity of the epithelial barrier [110]. Dysbiosis can produce microbial metabolites and directly influence immune cell activity, resulting in abnormal systemic immune responses that may initiate disease in individuals with a genetic predisposition. 

Comparative studies reveal significant differences in microbial taxa and metabolite profiles between RA patients and healthy controls [111]. One notable finding is the increased relative abundance of *Prevotella copri* in the gut microbiota in newly diagnosed RA patients [112,113]. This dysbiosis creates a pro-inflammatory environment by promoting the differentiation and expansion of Th17 cells within the intestinal lamina propria [113]. Additionally, an immunogenic protein derived from *Prevotella copri* has been linked to the breakdown of immune tolerance through a mechanism called molecular mimicry. This process can trigger Th1 cell responses and antibody production in certain RA patients [114]. In contrast, *Akkermansia muciniphila,* a bacterium that degrades mucin and is typically associated with enhanced mucosal barrier function and metabolic health, shows variable abundance in individuals with RA [115,116]. While its mucolytic activity can have both beneficial and adverse effects, its positive contributions are often related to strengthening tight junction integrity and producing SCFAs like acetate and propionate [115]. These metabolites are crucial for modulating T-cell responses and maintaining immune tolerance, potentially offering protection against inflammation [112,117]. Other important microbial metabolites, such as IPA, sphingomyelin species, argininosuccinate, glycine, and heptadecasphin-4-enine, have also been associated with either protective or pro-inflammatory roles in RA pathogenesis [118]. 

Microbiome-derived biomarkers are emerging as tools for diagnosis, disease monitoring, and therapeutic stratification (Table 5). Interventions targeting the microbiota, including dietary modulation and the use of specific probiotics or fecal microbiota transplantation, show promise as adjunctive strategies for RA management [119].

#### 3.1.7. Bioinformatics

Bioinformatics is a foundational pillar of contemporary RA research, providing the computational infrastructure to integrate and interrogate complex biological and clinical datasets. Through a systems biology framework, bioinformatics enables the fusion of multi-omics data, including genomics, transcriptomics, proteomics, epigenomics, and metabolomics, and microbiomics with clinical phenotypes. This integrative approach offers a multidimensional view of RA pathogenesis and facilitates the identification of robust biomarkers and actionable therapeutic targets [120,121,122,123,124,125].

**Multi-omics integration and predictive modeling:** Advanced bioinformatics pipelines harmonize heterogeneous omics datasets, allowing for the discovery of composite biomarkers for diagnosis, disease activity monitoring, and treatment response prediction [56,126,127]. Data fusion techniques are particularly valuable for constructing unified disease models that reflect RA’s molecular heterogeneity. ML and deep learning algorithms further enhance predictive modeling by extracting clinically relevant patterns from high-dimensional data. For instance, the Random Forest algorithm effectively integrates transcriptomic (RNA-Seq) and DNA methylation signatures from CD4+ T cells and monocytes [128]. This approach successfully predicts responses to biologics such as adalimumab (ADA) with approximately 86% accuracy and etanercept (ETN) with about 79% accuracy. It also identifies pathways, such as TNF signaling, that are enriched in ADA and ETN responders [128,129]. In the analysis of clinical data from thousands of RA patients, ML models like Lasso regression have been shown to outperform traditional methods in predicting the one-year persistence of methotrexate therapy [130]. Additionally, deep learning approaches, including Autoencoders, are being employed to combine high-dimensional multi-omics data to extract and define more homogeneous molecular subtypes of RA [131,132]. This work is paving the way for precise patient stratification.

**Pattern recognition and computational validation:** Advanced bioinformatics tools employ sophisticated pattern recognition and data mining algorithms to detect subtle regulatory perturbations in gene expression, miRNA profiles, and DNA methylation landscapes. These insights illuminate key pathogenic pathways [120,121,122,123]. Computational validation, through cross-referencing candidate biomarkers with curated databases and literature, is indispensable for ensuring reproducibility, robustness, and translational relevance before experimental validation.

**Data processing and visualization:** Initial data preprocessing and quality control are typically conducted using established platforms such as Bioconductor, FastQC, and DeepVariant. However, the increasing scale and complexity of RA datasets necessitate the adoption of ML- and artificial intelligence (AI)-driven analytical frameworks capable of extracting clinically actionable insights [14]. Visualization tools, such as heatmaps, network graphs, and dimensionality-reduction plots, play a vital role in translating complex data into interpretable formats for interdisciplinary collaboration.

These computational approaches are vital for advancing precision medicine in RA, offering scalable solutions for early diagnosis, patient stratification, and individualized therapeutic interventions. 

#### 3.1.8. Summary

**Figure 1:**
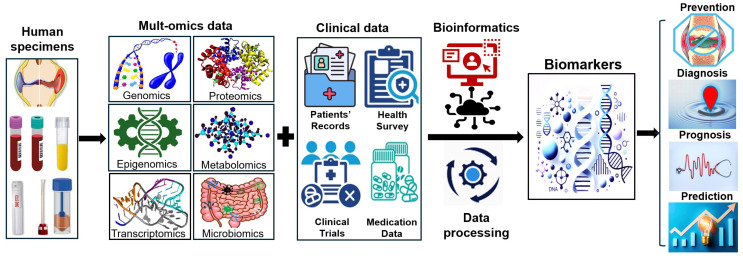
The diagram of biomarker discovery and biomarker application in rheumatoid arthritis.

The integration of multi-omics datasets with advanced bioinformatics frameworks is reshaping RA research. This system-level approach enables a deeper understanding of RA pathogenesis by linking molecular data, including genomics, transcriptomics, proteomics, metabolomics, and epigenomics, with clinical phenotypes. For example, the integration of plasma proteomics and metabolomics with clinical data allows for the identification of distinct molecular signatures in ACPA-negative RA patients, which are often poorly defined by traditional methods. This process reveals unique immune and metabolic pathways that differentiate them from ACPA-positive patients and can be used to refine diagnostic and stratification strategies [133]. Analyzing baseline transcriptomic and proteomic profiles (e.g., specific genes and protein levels in peripheral blood cells) can predict which patients will be non-responders to an anti-TNF drug [134]. This allows clinicians to switch patients immediately to a more effective therapy, preventing joint damage and unnecessary drug exposure [134]. During this integration, bioinformatics plays a pivotal role in harmonizing these complex datasets, facilitating the identification of high-confidence biomarkers (Figure 1).

Despite its potential, the widespread adoption of multi-omics technologies faces significant challenges. One major barrier is the high cost of comprehensive multi-omics testing, which particularly affects its implementation in routine clinical settings. To overcome this issue, innovations are needed in assay miniaturization, cost-effective sequencing platforms, and the strategic selection of omics layers based on their clinical utility. Moreover, the complexity of data analysis requires robust computational infrastructure and interdisciplinary expertise, highlighting the importance of developing scalable, user-friendly bioinformatics tools and standardized workflows. 

Currently, multi-omics approaches are mainly used in research environments to support hypothesis generation and biomarker discovery. However, commercial panels, especially in the fields of genomics and transcriptomics, are starting to emerge, providing targeted assays for clinical decision-making [135]. As analytical frameworks advance and costs decrease, the shift from research to clinical applications is expected to accelerate, opening the door to personalized medicine in RA.

#### 3.1.9. Molecular Signatures and Synovial Biopsy-Based Biomarkers

Persistent synovial inflammation is a defining feature of RA, and its direct interrogation is essential for elucidating disease mechanisms, refining patient stratification, and informing therapeutic decisions. The advent of minimally invasive ultrasound-guided biopsy has enabled consistent acquisition of high-fidelity synovial tissue [136], catalyzing synovial biomarker discovery.

Advanced molecular profiling techniques, including bulk and single-cell transcriptomics, proteomics, and spatial cellular analyses, have delineated distinct synovial pathotypes. The lympho-myeloid subtype, characterized by dense immune cell infiltration, correlates with aggressive joint damage [52] and increased reliance on bDMARDs [137]. Conversely, the fibroid/pauci-immune pathotype exhibits poor responsiveness to TNFi such as certolizumab-pegol [138], underscoring the need for tailored therapeutic strategies.

Biopsy-driven molecular stratification has demonstrated clinical utility. In TNFi-refractory RA, patients with low B-cell molecular signatures responded preferentially to tocilizumab over rituximab [139]. Transcriptomic analyses further identified a fibroblast-enriched signature predictive of rituximab resistance [140], highlighting the role of stromal cell phenotypes in treatment failure. 

Integrative studies, notably by Zhang et al., have mapped the synovial cellular landscape using multi-modal approaches, identifying 18 immune and stromal populations [141], and constructing a comprehensive single-cell atlas from over 314,000 cells [142]. Reduced PD-1^hi^CXCR5- peripheral helper T-cell frequencies in the synovium were associated with favorable TNFi responses [143], suggesting novel predictive biomarkers. 

These insights affirm synovial tissue as a critical reservoir for precision medicine in RA. Integrating synovial and peripheral immune profiling holds the potential to refine therapeutic strategies and accelerate the development of biomarker-driven treatments. However, the successful integration of synovial biopsy in both clinical and research settings relies on overcoming several critical procedural challenges. One major issue is accessibility; the technique requires specialized equipment and skilled personnel, which limits its availability to a small number of medical centers. Therefore, it is essential to standardize biopsy protocols, including site selection, tissue handling, and analytical methods, to ensure reproducibility and comparability across different studies and institutions. Furthermore, improving the consistency of procedures is crucial for effective long-term monitoring and comparative analyses, especially in clinical trials assessing therapeutic responses. Advancements in these areas are vital for optimizing the use of synovial tissue in research and clinical applications for RA.

## 4. Challenges and Future Directions of Biomarkers in RA

The incorporation of biomarkers into the care of RA represents a major step forward in precision medicine. However, the molecular and clinical diversity associated with RA complicates patient classification and reduces the predictive power of individual biomarkers. To address this challenge, multi-omics approaches and synovial biopsy generate high-dimensional datasets that require advanced computational tools, particularly ML and AI, to identify actionable biomarkers and clarify disease mechanisms. The integration of multi-omics data and AI-driven analysis is not merely a trend; it represents a critical evolution in the development of personalized care for RA. Looking forward, several innovative directions show promise for transforming RA management. Liquid biopsy methods, such as blood-based assays [144], may provide non-invasive alternatives to synovial tissue profiling, enhancing accessibility and facilitating dynamic disease monitoring. Furthermore, the integration of multi-omics data into digital twin models [145] could allow for the simulation of individual treatment responses, enabling clinicians to evaluate therapeutic strategies in a virtual environment before actual implementation. These advancements, coupled with improvements in wearable biosensors, real-time data analytics, and cloud-based diagnostic platforms, are poised to reshape the landscape of RA care and accelerate the realization of precision medicine.

Despite promising discoveries, translation into clinical practice remains challenging. Assay validation, cross-platform standardization, and reproducibility across diverse populations are essential for regulatory approval and clinical adoption. Interdisciplinary collaboration among clinicians, data scientists, and regulatory bodies is vital to overcoming translational barriers.

To bridge the gap between research and routine care, it is essential to focus on technology translation. This includes conducting thorough cost-effectiveness analyses to demonstrate the clinical value of biomarker-driven approaches, particularly in resource-constrained settings. Establishing standardized protocols for sample collection, data processing, and interpretation will enhance reproducibility and facilitate collaboration across multiple centers. Additionally, developing automated analysis platforms that can integrate multi-omics data with clinical parameters will streamline workflows and reduce the burden on healthcare providers. Equally important is the careful integration of these technologies into existing clinical pathways, ensuring that diagnostic innovations enhance rather than disrupt current practices. Future priorities include developing personalized biomarker panels, embedding AI-driven analytics into clinical workflows, and establishing interoperable diagnostic platforms. Addressing economic and logistical hurdles, such as assay cost, regulatory complexity, and clinician education, is critical for widespread implementation. Advancing these strategies will enable biomarker-guided care that supports earlier diagnosis, individualized treatment selection, and dynamic disease monitoring, ultimately improving outcomes for patients with RA.

## Figures and Tables

**Table 1:** The key autoantibodies in rheumatoid arthritis (RA), along with their sensitivity, specificity, prevalence in other conditions, clinical applications, and typical algorithms used.

Autoantibodies	Sensitivity	Specificity	Presence in Other Conditions	Clinical Application in RA	Typical Algorithms Used
Rheumatoid Factor	60–80%	70–80%	Healthy individuals, Sjögren’s syndrome, lupus, chronic infections	Diagnostic and prognostic marker, prediction of treatment responses to rituximab, sarilumab, tofacitinib	Ensemble Tree-Based Models, Multivariate Logistic Regression, Feature Importance Algorithms, ACR/EULAR Classification Criteria (2010)
Anti-Citrullinated Protein Antibodies	60–70%	>95%	Rarely in other conditions	Diagnostic and prognostic marker, prediction of treatment responses to rituximab, sarilumab, tofacitinib	Classification Algorithms, Receiver Operating Characteristic (ROC) Analysis, Random Forest, Feedforward Neural Networks, ACR/EULAR Classification Criteria (2010)
Anti-Carbamylated Protein Antibodies	35–50%	~90%	Rarely in other conditions	Useful for the diagnosis of seronegative RA, associated with a more aggressive disease	Multivariate Logistic Regression, Meta-Analysis.
Anti-Peptidyl Arginine Deiminase 4	20–30%	>90%	Rarely in other conditions	Associated with a more severe and erosive disease; may predict the need for biologic treatments	ROC Analysis, Correlation Analysis, Simple Classification Algorithms
Anti-Sa Antibodies	20–30%	>95%	Rarely in other conditions	Prediction of disease severity and joint erosions	Multivariate Regression Models, Correlation Analysis
Anti-Mutated Citrullinated Vimentin antibodies	70–80%	85–95%	Rarely in other conditions	Highly useful for the diagnosis of seronegative RA, associated with disease activity and severity	ROC Analysis, Classification Algorithms
Anti-Nuclear Antibodies	20–30%	30–50%	Lupus, Sjögren’s syndrome, Scleroderma	The presence may indicate an overlap with another autoimmune disease	Artificial Intelligence /Machine Learning models
Anti-Ro/SSA Antibodies	3–15%	30–50%	Sjögren’s syndrome, Lupus, Systemic sclerosis	The presence may indicate an overlap with Sjögren’s syndrome	Clustering Algorithms
Anti-La/SSB Antibodies	3–10%	30–50%	Sjögren’s syndrome, Lupus, Systemic sclerosis	The presence usually indicates an overlap with Sjögren’s syndrome	Clustering Algorithms

**Table 2:** Key inflammatory biomarkers in rheumatoid arthritis (RA), including their sensitivity, specificity, presence in other conditions, clinical applications, and typical algorithms used.

Inflammatory Biomarkers	Sensitivity	Specificity	Presence in Other Conditions	Clinical Application in RA	Typical Algorithm
CRP	40–60%	~40%	Elevated in a wide range of inflammatory, infectious, and tissue-damaging conditions	A key component of disease activity scores, monitoring disease activity and treatment response	DAS28-CRP, ACR/EULAR Classification Criteria (2010)
ESR	40–60%	~40%	Elevated in many inflammatory and infectious conditions, and certain cancers	A key component of disease activity scores, monitoring disease activity and treatment response	DAS28-ESR, ACR/EULAR Classification Criteria (2010)
SAA	40–60%	40–60%	Elevated in various inflammatory conditions	Monitoring disease activity, predicting treatment response	Multi-Biomarker Disease Activity (MBDA) Score
MMP-3	40–80%	50–70%	Osteoarthritis, other joint diseases, and lupus	A marker of joint destruction and cartilage breakdown	MBDA Score
Calprotectin	60–80%	~90%	Inflammatory bowel disease, psoriatic arthritis, and infections	Correlated with disease activity, predicting a poor radiographic outcome	Receiver Operating Characteristic (ROC) Analysis, MBDA Scores
14-3-3η	60–70%	80–90%	Osteoporosis, other autoimmune diseases	Early diagnostic and prognostic marker, predicting a more erosive disease	ROC Analysis
TNF-α	40–70%	40–60%	Various autoimmune /inflammatory diseases	A key therapeutic target, monitoring disease activity	MBDA Score
IL-6	40–70%	40–60%	Various autoimmune/inflammatory diseases	A key therapeutic target, monitoring disease activity	MBDA Score

CRP: C-reactive protein. ESR: Erythrocyte sedimentation rate. IL: Interleukin. MMP: Matrix Metalloproteinase. SAA: Serum amyloid A. TNF: Tumor necrosis factor.

**Table 3:** Key genetic biomarkers in rheumatoid arthritis (RA), including their sensitivity, specificity, presence in other conditions, clinical applications, and typical algorithms used.

Genetic Biomarkers	Sensitivity	Specificity	Presence in Other Conditions	Clinical Application in RA	Typical Algorithm
*HLA-DRB1*	80–90%	60–70%	A key shared risk factor for multiple autoimmune diseases	May be used for risk assessment and early diagnosis	Polygenic Risk Scores (PRS) Models
*PTPN22*	50–60%	60–80%	A key shared risk factor for multiple autoimmune diseases	Potential use in predicting disease risk and tailoring treatment	Machine learning (ML) Models (e.g., Logistic Regression, Decision Trees, XGBoost)
*STAT4*	50–70%	40–60%	Associated with a variety of autoimmune and inflammatory conditions	Limited clinical application, could be a potential target for new therapies	PRS / ML Models
*TRAF1/C5*	40–60%	40–60%	Associated with a variety of autoimmune and inflammatory conditions	Not used in clinical practice for diagnosis or prognosis	PRS Models
*PADI4*	~70%	40–60%	Rarely in other conditions	Potential use for predicting a more aggressive and erosive disease	ML Models
*TNFAIP3*	40–60%	40–60%	Associated with numerous other autoimmune conditions	Not used in clinical practice for diagnosis or prognosis	ML Models
*IL-2RA*	40–50%	40–50%	Associated with numerous other autoimmune conditions	Not used in clinical practice for diagnosis or prognosis	ML Models
*CD40*	40–60%	40–60%	Associated with numerous other autoimmune conditions	Not used in clinical practice for diagnosis or prognosis	PRS Models
*CTLA4*	40–70%	40–60%	A well-known risk factor for numerous autoimmune diseases	Not used in clinical practice for diagnosis or prognosis	ML Models

**Table 4:** Key metabolite biomarkers in rheumatoid arthritis (RA), along with their sensitivity, specificity, presence in other conditions, potential clinical applications, and typical algorithms.

Metabolite Biomarkers	Sensitivity	Specificity	Presence in Other Conditions	Clinical Application in RA	Typical Algorithm Used
Glyceric Acid	Limited data available	Limited data available	Glyceric Aciduria, some cardiovascular diseases	A potential marker for disease activity	Machine Learning (ML) Classifiers (e.g., Random Forest, Logistic Regression)
Lactic Acid	30–40%	30–50%	Elevated in a wide range of conditions	A general marker for increased tissue inflammation	Statistical Analysis (Correlation/Regression), ML
3-Hydroxyisovaleric Acid	Limited data available	Limited data available	Leucine deficiency and other metabolic disorders	Not used clinically for diagnosis or monitoring.	ML Classifiers
Angiotensinogen	40–60%	40–60%	Elevated in hypertension and metabolic syndrome	A potential diagnostic marker for seronegative RA	Statistical Analysis, ML
Serum Amyloid A-4 Protein	Limited data available	Limited data available	Elevated in various inflammatory conditions	A potential prescreening marker when used in combination with other markers	Statistical Analysis, ML
Vitamin D-Binding Protein	Limited data available	Limited data available	Liver disease, kidney disease, and sepsis	A component of a multi-biomarker panel for the diagnosis of seronegative RA	Statistical Analysis, ML
Retinol-Binding Protein-4	40–60%	40–60%	Metabolic syndrome and cardiovascular diseases	A potential component of a diagnostic panel for seronegative RA	Statistical Analysis, ML

**Table 5:** Key microbiota biomarkers in rheumatoid arthritis (RA), along with their sensitivity, specificity, observed changes in RA and other conditions, potential clinical applications, and typical algorithms used.

Microbiota Biomarkers	Sensitivity	Specificity	Changes in RA	Changes in Other Conditions	Clinical Application for RA	Typical Algorithm Used
*Prevotella copri*	70%	~70%	**↑**	Inflammatory bowel disease, psoriatic arthritis, and other autoimmune diseases	A diagnostic marker for new-onset RA, predict response to MTX therapy	Statistical Analysis, Linear Discriminant Analysis, Effect Size, Machine Learning (ML) Classifiers (Random Forest)
*Collinsella*	30–50%	30–50%	**↑**	Psoriasis, ankylosing spondylitis, and other spondyloarthropathies	Associated with high ACPA levels, used to understand pathogenesis	Differential Abundance Analysis (DAA), Correlation Analysis
*Lactobacillus*	Varies	Varies	**↓**	Inflammatory bowel disease, metabolic disorders, allergies, and cardiovascular disease	Can be used for potential probiotic interventions	DAA, Aitchison Distance (Beta-diversity)
*Bacteroides*	20–50%	20–50%	**↓**	Obesity, diabetes, and Inflammatory bowel disease	A component of a predictive model, associated with a poorer response to MTX	Statistical Analysis, Regression Models
*Faecalibacterium*	20–50%	20–50%	**↓**	Inflammatory bowel diseases and chronic fatigue syndrome	A general marker of dysbiosis, a potential probiotic treatment target	Statistical Analysis, Functional Prediction Tools (e.g., PICRUSt or Tax4Fun)
*Eggerthellales*	20–50%	20–50%	**↑**	Some species are associated with gut infections and inflammation	A potential marker for disease severity and a potential probiotic treatment target	DAA, Correlation Analysis
*Enterococcus*	20–50%	20–50%	**↓**	A wide range of infections, including urinary tract infections	The general decrease is a marker of dysbiosis	DAA, Principal Coordinate Analysis/Non-metric Multidimensional Scaling
*Bifidobacterium species*	20–50%	20–50%	**↓**	Depleted in various inflammatory and metabolic diseases	Monitoring gut health and potential probiotic treatments	Statistical Analysis, Correlation Analysis

MTX: methotrexate; ACPA: anti-citrullinated protein antibodies. **↓** indicates decrease and **↑** increase.

## Data Availability

No new data were created or analyzed in this study.

## Abbreviations

The following abbreviations are used in this manuscript:

RA	Rheumatoid arthritis
RF	Rheumatoid factor
ACPA	Anti-citrullinated protein antibodies
cs/bDMARDs	Conventional synthetic/biologic disease-modifying antirheumatic drugs
anti-CarP	Anti-carbamylated protein
anti-PAD4	Anti-peptidyl arginine deiminase 4
CRP	C-reactive protein
ESR	Erythrocyte sedimentation rate
SAA	Serum amyloid A
MMP	Matrix metalloproteinase
IL	Interleukin
TNF	Tumor necrosis factor
SNP	Single-nucleotide polymorphism
HDAC	Inhibition of histone deacetylase
ML	Machine learning
NGS	Next-generation sequencing
GWAS	Genome-wide association studies
SCFA	Short-chain fatty acid
VDBP	Vitamin D binding protein
IPA	Indole-3-propionate
